# Cycloastragenol Provides Pharmacokinetic Advantages over Astragaloside IV with Enhanced Cochlear Exposure and Protection Against Age-Associated Hearing Dysfunction

**DOI:** 10.3390/pharmaceutics18081037

**Published:** 2026-08-20

**Authors:** Yaqian Gao, Yiheng Liang, Haiyan Chen, Zigui Wang, Yanchang Huang, Wei Zhou, Zhiyun Du

**Affiliations:** School of Biomedical and Pharmaceutical Sciences, Guangdong University of Technology, Guangzhou 510006, China

**Keywords:** cycloastragenol, Astragaloside IV, pharmacokinetics, active metabolite, cochlear exposure, tissue distribution, age-related hearing loss, hair cell protection

## Abstract

**Background:** Adequate inner-ear exposure is a key challenge in treating age-related hearing loss (ARHL). Astragaloside IV (AS-IV), the principal saponin of Astragalus membranaceus, has anti-aging activity but poor oral bioavailability; its aglycone cycloastragenol (CAG) may represent a pharmacokinetically optimized active moiety. **Methods**: We compared the pharmacokinetics, cochlear exposure, and biotransformation of CAG and AS-IV after oral dosing in mice by LC-MS/MS, and evaluated CAG in a D-galactose-induced accelerated-aging rat model (ABR, hair-cell morphology, redox and cytokine assays) and in D-galactose-stressed HEI-OC1 cells. **Results**: CAG achieved markedly higher plasma and perfused whole-cochlea exposure than intact AS-IV after equivalent dosing, even after molar-dose normalization. AS-IV was biotransformed to CAG in vivo and in liver microsomes, supporting CAG as a quantitatively important active metabolite. In rats, oral CAG reduced ABR threshold elevation and preserved hair-cell architecture, improved cochlear redox status (higher SOD/GSH; lower ROS/MDA), and lowered serum TNF-α/IL-6. In HEI-OC1 cells, CAG attenuated mitochondrial membrane-potential loss and apoptosis. **Conclusions**: At equimolar concentrations, CAG and AS-IV showed comparable protective activity in vitro, indicating that the advantage of CAG resides mainly in its pharmacokinetic profile. CAG thus attenuates auditory dysfunction in an accelerated-aging model with superior exposure, supporting its further development for age-associated hearing disorders.

## 1. Introduction

Age-related hearing loss (ARHL), or presbycusis, is a progressive sensory disorder associated with impaired communication, social isolation, depression, and cognitive decline. Its pathogenesis is multifactorial and includes cumulative oxidative stress, mitochondrial dysfunction, chronic low-grade inflammation, cellular senescence, and loss of cochlear sensory and neural elements. Hearing aids and cochlear implants improve communication for many patients but do not directly prevent the underlying biological degeneration. Pharmacological strategies that preserve auditory function therefore remain an important unmet need [1,2,3,4,5].

**Scheme 1:**
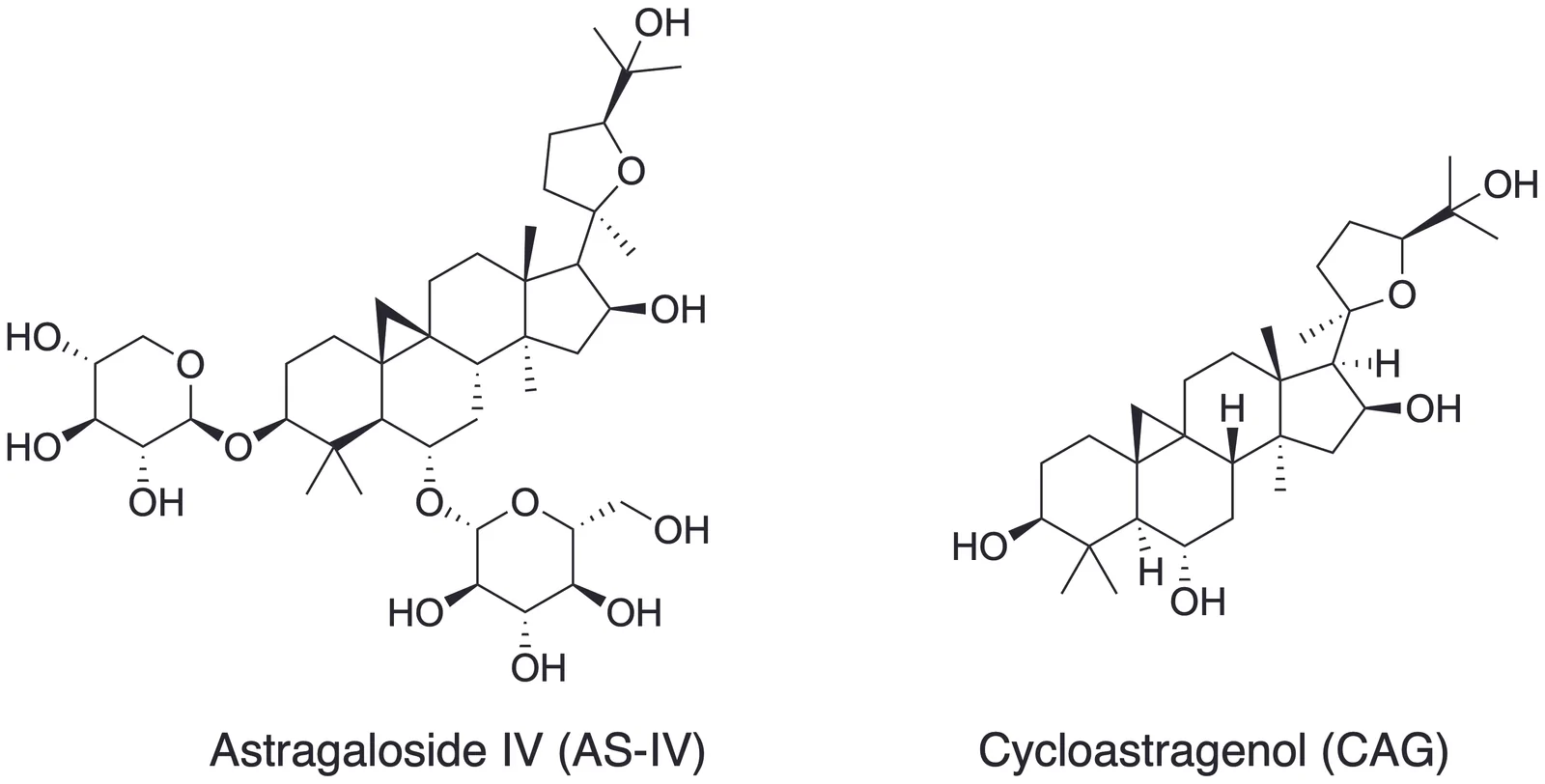
Chemical structure of astragaloside IV (AS-IV) and cycloastragenol (CAG).

Systemically administered compounds must achieve adequate exposure in the inner ear, where the blood-labyrinth barrier regulates exchange between the circulation and cochlear tissues. Drug exposure depends on multiple factors, including absorption, metabolism, plasma disposition, physicochemical properties, and tissue transport [6,7]. Natural products derived from Astragalus membranaceus have garnered significant attention for their geroprotective properties. Astragaloside IV (AS-IV, Scheme 1), the primary bioactive saponin of Astragalus, has demonstrated notable anti-apoptotic and antioxidant capabilities in various in vitro models [8,9,10,11]. However, AS-IV is a highly glycosylated and relatively hydrophilic molecule with limited oral exposure, and the extent to which intact AS-IV or its metabolites reach cochlear tissues remains incompletely defined [12,13,14]. These uncertainties motivate direct comparison with its less glycosylated aglycone.

Cycloastragenol (CAG, Scheme 1), the aglycone of AS-IV, has lower molecular weight and fewer polar sugar groups, properties that could alter absorption, metabolism, and tissue distribution [15,16,17,18]. Stripped of its polar sugar moieties, CAG possesses a significantly lower molecular weight, a more compact molecular architecture, and optimized lipophilicity. CAG has also been investigated in aging- and stress-related experimental systems [19,20,21,22,23]. SIRT1 is biologically relevant to cochlear aging because prior studies link it causally to oxidative-stress responses, mitochondrial quality control, senescence, and hair-cell survival [24,25,26,27]. Whether CAG directly engages SIRT1 in auditory tissues, however, has not been established.

We hypothesized that direct CAG administration would produce greater systemic and cochlear exposure than AS-IV administration and would attenuate auditory dysfunction in a D-galactose-induced accelerated-aging model. We therefore combined auditory functional and morphological assessments with comparative in vivo pharmacokinetic profiling and ex vivo biotransformation assays. Redox-, inflammation-, mitochondrial-, and apoptosis-related outcomes were evaluated in animal tissues and in vitro cellular models. Systems pharmacology and molecular docking were used to nominate candidate pathways and protein interactions for future causal testing.

## 2. Materials and Methods

### 2.1. Materials and Cell Culture

Dulbecco’s modified Eagle’s medium (DMEM; Servicebio, Wuhan, China) supplemented with fetal bovine serum (FBS; Daxi Bio, Hohhot, China) and penicillin–streptomycin (Servicebio, Wuhan, China) was used for cell culture. D-Galactose was purchased from Shanghai Macklin Biochemical Co., Ltd. (Shanghai, China). Astragaloside IV (AS-IV) was obtained from Servicebio (Wuhan, China), and cycloastragenol (CAG) was purchased from Shanghai Aladdin Biochemical Technology Co., Ltd. (Shanghai, China). Dimethyl sulfoxide (DMSO; Dalian Meilun Biotechnology Co., Ltd., Dalian, China), 0.25% trypsin (Servicebio, Wuhan, China), and the Cell Counting Kit-8 (CCK-8; Suzhou NCM Biotech Co., Ltd., Suzhou, China) were used for cell culture and viability assays. The fluorescent probes 2′,7′-dichlorodihydrofluorescein diacetate (DCFH-DA), JC-1, and Hoechst 33,342 were purchased from Merck KGaA (Darmstadt, Germany). The Annexin V-FITC apoptosis detection kit was obtained from Beyotime Biotechnology (Shanghai, China). Phosphate-buffered saline (PBS; Servicebio, Wuhan, China) was used for washing and sample preparation. All other reagents were of analytical grade.

Erlong Zuoci Wan (Erlong Zuoci decoction) is a classical traditional Chinese medicine formula for tinnitus and hearing loss, derived from Liuwei Dihuang Wan with the addition of Magnetitum (Ci Shi) and Bupleuri Radix (Chai Hu), and comprises eight herbs (Rehmanniae Radix Praeparata, Corni Fructus, Dioscoreae Rhizoma, Poria, Alismatis Rhizoma, Moutan Cortex, Magnetitum, and Bupleuri Radix) [28]. It was purchased from Beijing Tongrentang Co., Ltd. (Beijing, China).

The murine auditory cell line HEI-OC1 was purchased from the House Ear Institute (Los Angeles, CA, USA). The cells were cultured in high-glucose Dulbecco’s Modified Eagle Medium (DMEM; Servicebio, Wuhan, China) supplemented with 10% fetal bovine serum (FBS; Daxi Bio, Kunming, China) and 1% penicillin–streptomycin (Servicebio, Wuhan, China), and were maintained at 33 °C in a humidified atmosphere containing 10% CO_2_ (permissive conditions) [29]. Under these conditions, the cells exhibited an epithelial-like, polygonal (cobblestone) morphology, consistent with the undifferentiated auditory progenitor phenotype. Cells between passages 15 and 25 were used for all experiments.

### 2.2. Senescence Model and Cell Viability Assay

Cell viability was evaluated using the Cell Counting Kit-8 (CCK-8) assay. The Cell Counting Kit-8 (CCK-8; NCM Biotech, Suzhou, China) is a colorimetric cell-viability assay based on the water-soluble tetrazolium salt WST-8 [2-(2-methoxy-4-nitrophenyl)-3-(4-nitrophenyl)-5-(2,4-disulfophenyl)-2H-tetrazolium, monosodium salt]. In viable cells, intracellular dehydrogenases reduce WST-8, in the presence of the electron mediator 1-methoxy-5-methylphenazinium methyl sulfate (mPMS), to an orange water-soluble formazan product, and the absorbance measured at 450 nm is directly proportional to the number of viable cells [30].

HEI-OC1 cells were seeded into 96-well plates at a density of 1 × 10^4^ cells/well and cultured for 24 h. To screen for the optimal senescence-inducing concentration, cells were exposed to varying concentrations of D-galactose (D-gal) (10, 20, 30, 40, 50, 60, 70, and 80 mg/mL) for 24 h, with 6 replicate wells per group.

For the drug screening and protection assays, cells were divided into the following groups: Control group, Model group (40 mg/mL D-gal), positive control group (40 mg/mL D-gal + 500 μg/mL Erlong Zuoci Wan), AS-IV group (40 mg/mL D-gal + 50 μM Astragaloside IV), and CAG groups (40 mg/mL D-gal + 10, 25, or 50 μM Cycloastragenol). The CAG concentrations were selected on the basis of a pilot concentration–response experiment in HEI-OC1 cells, in which 50 μM CAG increased cell viability by 12.33% relative to the control, whereas concentrations of 100 μM and above were cytotoxic. AS-IV was used at 50 μM to provide an equimolar comparison with the optimal CAG concentration. The Control group comprised HEI-OC1 cells cultured under normal conditions in the absence of D-galactose and test compounds, and served as the untreated reference. The Erlong Zuoci Wan concentration (500 μg/mL) was selected with reference to published in vitro studies of Erlong Zuoci Wan extracts in auditory cell models [31]. Following a 24 h co-incubation, CCK-8 solution was added to each well, and the absorbance was measured using a Varioskan LUX microplate reader (Thermo Fisher Scientific, Waltham, MA, USA) to calculate the cell survival rate.

To verify the successful establishment of the senescence model at the molecular level, the mRNA expression of the senescence markers CDKN2A (encoding p16^INK4a^, referred to as P16) and CDKN1A (encoding p21^CIP1/WAF1^, referred to as P21) was quantified. HEI-OC1 cells were treated with 40 mg/mL D-galactose for 24 h. Total RNA was extracted using an RNA extraction kit (Servicebio, Wuhan, China), and complementary DNA was synthesized by reverse transcription (Servicebio, Wuhan, China). Real-time PCR was performed on a Bio-Rad CFX96 system (Bio-Rad Laboratories, Hercules, CA, USA) using SYBR Green master mix (Servicebio, Wuhan, China) with the following primers: CDKN2A forward 5′-CGAACTCTTTCGGTCGTACCC-3′ and reverse 5′-CGAATCTGCACCGTAGTTGAGC-3′; CDKN1A forward 5′-CCTGGTGATGTCCGACCTG-3′ and reverse 5′-CCATGAGCGCATCGCAATC-3′ [32]; and β-actin (Actb; internal reference) forward 5′-GGCTGTATTCCCCTCCATCG-3′ and reverse 5′-CCAGTTGGTAACAATGCCATGT-3′. The cycling conditions were 95 °C for 30 s, followed by 40 cycles of 95 °C for 5 s and 60 °C for 30 s, with a final melt-curve step. Relative mRNA expression was calculated using the 2^−ΔΔCt^ method [33] with β-actin as the internal reference, normalized to the untreated Control group. The experiments were conducted in accordance with the MIQE guidelines.

### 2.3. ROS, MMP, and Apoptosis Analyses

The accumulation of intracellular ROS was detected using flow cytometry with the fluorescent probe 2′,7′-dichlorodihydrofluorescein diacetate (DCFH-DA, Beyotime, Shanghai, China). HEI-OC1 cells were seeded into 6-well plates at a density of 2 × 10^5^ cells/well and cultured for 24 h. After establishing the experimental groups (Control, Model, Erlong Zuoci Wan, 50 μM AS-IV, and 10/25/50 μM CAG) and co-incubating with 40 mg/mL D-gal for 24 h, the cells were harvested and incubated with 5 μM DCFH-DA for 30 min in the dark. The fluorescence intensity, indicative of ROS levels, was subsequently measured and quantified using an Attune NxT flow cytometer (Thermo Fisher Scientific, Waltham, MA, USA).

The changes in mitochondrial membrane potential were assessed using the JC-1 fluorescent probe, a lipophilic cationic dye that shifts its fluorescence emission depending on the membrane potential state [34]. HEI-OC1 cells were cultured in confocal dishes and subjected to the designated treatments (Control, 40 mg/mL D-gal, Erlong Zuoci Wan, 50 μM AS-IV, and 10/25/50 μM CAG) for 24 h. Following treatment, the cells were washed and incubated with JC-1 staining solution in the dark at 33 °C. Cell nuclei were counterstained with Hoechst 33,342. The cells were then washed with JC-1 buffer and immediately observed under a fluorescence microscope (EVOS M7000 cell imaging system; Thermo Fisher Scientific, Waltham, MA, USA). In healthy cells with high MMP, JC-1 accumulates in the mitochondrial matrix to form J-aggregates, emitting red fluorescence; in cells with depolarized mitochondria, JC-1 remains in the cytoplasm as monomers, emitting green fluorescence. The collapse of MMP was evaluated by monitoring the shift from red to green fluorescence.

Cellular apoptosis was quantified using the Annexin V-FITC/Propidium Iodide (PI) Apoptosis Detection Kit (Beyotime, Shanghai, China). During early apoptosis, phosphatidylserine (PS) translocates from the inner to the outer leaflet of the plasma membrane, which can be specifically labeled by Annexin V-FITC. PI is a membrane-impermeable dye that exclusively stains the DNA of late apoptotic or necrotic cells with compromised membrane integrity [35].

HEI-OC1 cells were seeded in 6-well plates and treated according to the established experimental groups for 24 h. After the co-incubation period, cells were harvested, washed with cold PBS, and gently resuspended in 1× binding buffer. The cell suspension was then double-stained with Annexin V-FITC and PI solutions, gently vortexed, and incubated in the dark at room temperature. Following incubation, the samples were immediately analyzed using a flow cytometer. The cell populations were categorized as viable cells (Annexin V−/PI−), early apoptotic cells (Annexin V+/PI−), and late apoptotic/necrotic cells (Annexin V+/PI+). The total apoptosis rate for each group was calculated as the sum of the early and late apoptotic cell percentages.

### 2.4. Western Blot Analysis

The expression levels of key apoptosis-related proteins were evaluated using Western blot analysis. HEI-OC1 cells were seeded into 6-well plates at a density of 2 × 10^5^ cells/well and incubated for 24 h to allow for attachment. Following the experimental design, cells were treated with the designated regimens (Control, Model with 40 mg/mL D-gal, Erlong Zuoci Wan at 500 μg/mL, AS-IV at 50 μM, and CAG at 10, 25, or 50 μM) for 24 h.

Following treatment, the cells were harvested, and total protein was extracted using RIPA lysis buffer. The protein concentration of each sample was accurately determined using a BCA protein assay kit. Equivalent amounts of denatured protein samples were separated by sodium dodecyl sulfate-polyacrylamide gel electrophoresis (SDS-PAGE) using an SVT-2 electrophoresis apparatus (Servicebio, Wuhan, China) and subsequently transferred onto polyvinylidene fluoride (PVDF) membranes.

To prevent non-specific binding, the membranes were blocked with non-fat milk at room temperature. The membranes were then incubated overnight at 4 °C with the following primary antibodies: anti-Bax (1:2000), anti-Bcl-2 (1:4000), anti-Cleaved Caspase-3 (1:2000), anti-Caspase-3 (1:2000), and anti-β-actin (1:15,000), which served as the internal loading control. After rigorous washing, the membranes were incubated with a goat anti-rabbit IgG secondary antibody (1:15,000) at room temperature. The protein bands were visualized using an enhanced chemiluminescence (ECL) detection system, and the grey values (optical density) of the target bands were quantified and analyzed using ImageJ software (v1.8.0, National Institutes of Health, Bethesda, MA, USA). The relative protein expression levels were normalized to β-actin.

### 2.5. Animal Model and Drug Administration

Eight-week-old male Sprague-Dawley (SD) rats weighing 250 ± 5 g were housed under controlled conditions (22 ± 2 °C, 50 ± 10% relative humidity, 12 h light/dark cycle) with ad libitum access to food and water. After acclimatization, the rats were randomly allocated to five groups (n = 6 per group). Accelerated-aging auditory dysfunction was induced by daily intraperitoneal administration of D-galactose (1000 mg/kg/day) for 8 consecutive weeks.

The groups were Control, D-gal model, Erlong Zuoci Wan positive control (GP), low-dose CAG (CAG-L), and high-dose CAG (CAG-H). After the 8-week modeling period, the GP group received Erlong Zuoci Wan (100 mg/kg/day, orally), and the CAG-L and CAG-H groups received CAG at 5 and 20 mg/kg/day, respectively, by oral gavage for 4 consecutive weeks. CAG was suspended in 0.5% sodium carboxymethyl cellulose (CMC-Na). During the treatment period, the Control group received no D-galactose and was administered the vehicle (0.5% CMC-Na) by oral gavage during the treatment period; the Model group received D-galactose (1000 mg/kg/day, i.p.) followed by the vehicle. The D-galactose dose and treatment duration were selected based on established accelerated-aging protocols [36], and the CAG doses (5 and 20 mg/kg/day) were selected based on our preliminary studies and in vitro concentration-response experiments.

### 2.6. Auditory Brainstem Response (ABR) Measurement

To evaluate auditory function, Auditory Brainstem Response (ABR) thresholds were recorded at baseline and at the study endpoint following 8 weeks of D-galactose modeling and 4 weeks of treatment [37]. The rats were anesthetized, and subdermal needle electrodes were placed at the vertex (active), the mastoid of the tested ear (reference), and the contralateral mastoid (ground). Tone bursts at 4, 8, 16, and 32 kHz were delivered through insert earphones. The ABR threshold was defined as the lowest stimulus intensity (dB SPL) that elicited a reproducible, visually distinguishable wave II or wave III.

### 2.7. Cochlear Surface Preparation and Hair Cell Quantification

To assess cochlear hair-cell morphology, the animals were euthanized by an intraperitoneal overdose of sodium pentobarbital combined with CO_2_ inhalation, in accordance with institutional animal care and use committee guidelines after the final ABR measurements, and the temporal bones were rapidly removed. The cochleae were fixed by perilymphatic perfusion of 4% paraformaldehyde in 0.1 M phosphate-buffered saline (PBS, pH 7.4) through the round window (approximately 500 μL over 5 min), followed by immersion in the same fixative overnight at 4 °C. After decalcification in 10% ethylenediaminetetraacetic acid (EDTA, pH 7.4) for 7 days at 4 °C, the cochleae were microdissected under a stereomicroscope, and the basilar membrane containing the organ of Corti was isolated from the middle turn. The specimens were stained with 0.5% silver nitrate (AgNO_3_) solution for 30 min in the dark and developed under bright light until the hair-cell boundaries were clearly delineated. After rinsing, the basilar membranes were flat-mounted on glass slides in glycerol [38].

The flat-mounted specimens were examined under a light microscope at ×400 magnification. Inner hair cells (IHCs) and outer hair cells (OHCs) were identified by their characteristic silver-stained outlines. A hair cell was counted as structurally intact only when its cell body showed a complete, clearly delineated boundary and an orderly position within the single IHC row or the three OHC rows; cells with missing, fragmented, or distorted outlines were scored as damaged or lost. For systematic quantification, the numbers of surviving (structurally intact) hair cells per 100 μm length of the basilar membrane were counted in three representative fields of the middle turn from one cochlea per animal (n = 6 rats per group), and the counts were expressed as the mean ± SD.

### 2.8. Cochlear Oxidative Stress and Serum Cytokines

Following the final auditory assessments and an overnight fast, the rats were deeply anesthetized by an intraperitoneal overdose of sodium pentobarbital combined with CO_2_ inhalation, in accordance with institutional animal care and use committee guidelines. Blood samples were collected from the abdominal aorta and centrifuged at 3000× *g* for 15 min at 4 °C to separate the serum (CF1524R refrigerated centrifuge; SCILOGEX, Rocky Hill, CT, USA). The obtained serum aliquots were immediately frozen and stored at −80 °C for subsequent inflammatory cytokine analysis. Subsequently, the animals were euthanized, and the temporal bones were rapidly removed. The cochleae were meticulously dissected on ice, and the isolated cochlear tissues were homogenized using a JXFSTPRP-48 tissue homogenizer (Shanghai Jingxin Industrial Development Co., Ltd., Shanghai, China) in ice-cold phosphate-buffered saline (PBS) or designated lysis buffers. The tissue homogenates were then centrifuged at 12,000× *g* for 15 min at 4 °C, and the resulting supernatants were collected. Total protein concentrations in the cochlear supernatants were determined using a BCA Protein Assay Kit to standardize all subsequent biochemical measurements.

The redox status within the cochlear microenvironment was evaluated by quantifying the levels of Reactive Oxygen Species (ROS,, Beyotime, Shanghai, China), Malondialdehyde (MDA), Superoxide Dismutase (SOD), and Glutathione (GSH) using corresponding commercial assay kits according to the manufacturers’ instructions. The accumulation of ROS in the cochlear tissue homogenates was measured using a specific ROS fluorescent probe (DCFH-DA), with the fluorescence intensity recorded via a multimode microplate reader. The concentration of MDA, a primary biomarker of lipid peroxidation, was assessed using the thiobarbituric acid (TBA) colorimetric method. Furthermore, the endogenous antioxidant capacity was evaluated by determining the specific activity of SOD and the content of GSH using precise colorimetric assay kits. All oxidative stress and antioxidant parameters were normalized to the corresponding total tissue protein concentration.

To evaluate the systemic inflammatory response, the concentrations of core pro-inflammatory cytokines in the peripheral circulation were quantified. The serum levels of Tumor Necrosis Factor-alpha (TNF-α) and Interleukin-6 (IL-6) were measured using specific rat commercial ELISA kits following the manufacturer’s rigorous protocols. Briefly, the diluted serum samples and standard solutions were added to appropriate microplate wells pre-coated with specific capture antibodies. Following sequential incubation and washing steps, enzyme-linked detection antibodies and colorimetric substrates were applied. The optical density (OD) was measured at 450 nm using a microplate reader, and the absolute concentrations of TNF-α and IL-6 were calculated by interpolating from their respective standard curves.

### 2.9. Pharmacokinetic Study and LC-MS/MS Analysis

To evaluate the systemic absorption and inner ear distribution profiles of the Astragalus-derived compounds, an in vivo pharmacokinetic (PK) study was conducted. Healthy male C57BL/6 mice were fasted overnight with free access to water prior to the experiment. The mice were randomly assigned to either the Astragaloside IV (AS-IV) group or the Cycloastragenol (CAG) group. Both compounds were freshly suspended in a 0.5% sodium carboxymethyl cellulose (CMC-Na) solution. The mice received a single oral dose of 20 mg/kg of either AS-IV or CAG via intragastric gavage.

A terminal sampling design was used. At 0.25, 0.5, 1, 2, 4, 8, 12, and 24 h after dosing, mice (n = 6 per treatment group per time point) were deeply anesthetized by an intraperitoneal overdose of sodium pentobarbital combined with CO_2_ inhalation, in accordance with institutional animal care and use committee guidelines. Whole blood was collected into heparinized tubes and centrifuged at 4000× *g* for 10 min at 4 °C to obtain plasma. The animals were then transcardially perfused with ice-cold physiological saline, after which both intact cochleae were rapidly collected, blotted dry, weighed, and homogenized in ice-cold physiological saline. Plasma and cochlear homogenates were stored at −80 °C until LC-MS/MS analysis.

The concentrations of the analytes in plasma and cochlear homogenates were quantified using a validated high-performance liquid chromatography-tandem mass spectrometry (LC-MS/MS) system. Given the potential in vivo biotransformation (deglycosylation) of the macromolecular saponin AS-IV into its active aglycone CAG, a high-sensitivity simultaneous detection method was established to quantify both AS-IV and CAG in the biological matrices. The samples underwent protein precipitation and extraction prior to injection into the LC-MS/MS system. Chromatographic separation and mass spectrometric detection in multiple reaction monitoring (MRM) mode were optimized to ensure high specificity and precision.

The drug concentration-time data were analyzed using non-compartmental analysis (NCA) with the pharmacokinetic software WinNonlin (Version 6.3, Pharsight Corporation, Mountain View, CA, USA). Key PK parameters, including maximum concentration (C_max_), time to reach maximum concentration (T_max_), half-life (t_1/2_), and area under the concentration-time curve (AUC), were calculated for the plasma profiles. Whole-cochlea concentration-time curves were plotted to characterize tissue exposure. The cochlea-to-plasma distribution coefficient (K_p_) was calculated as the ratio of cochlear AUC_0–24h_ to the matched plasma AUC_0–24h_ for each analyte. These measurements were not interpreted as direct evidence of BLB permeability.

To evaluate metabolic stability and AS-IV-to-CAG biotransformation, AS-IV and CAG were separately incubated with mouse liver microsomes (0.5 mg protein/mL) in 100 mM phosphate buffer (pH 7.4) containing 3 mM MgCl_2_ at 37 °C. After a 5 min preincubation, reactions were initiated by adding an NADPH-regenerating system (1 mM final concentration). Parallel controls omitted NADPH, used heat-inactivated microsomes, or contained no test compound. At 0, 15, 30, 45, and 60 min, aliquots were quenched with ice-cold acetonitrile containing an internal standard, vortexed, and centrifuged at 14,000× *g* for 10 min at 4 °C. Supernatants were analyzed by the validated LC-MS/MS method. AS-IV-derived CAG was identified by concordance with the CAG reference standard in chromatographic retention time and diagnostic MS/MS transitions. The natural logarithm of the percentage of parent compound remaining was plotted against incubation time to determine the elimination rate constant (*k*). Subsequently, the in vitro metabolic half-life (*t*_1/2_) and intrinsic clearance (*CL*_int_) were calculated to comprehensively profile the metabolic stability of the compounds.

### 2.10. Systems Pharmacology and Molecular Docking

To elucidate the multi-target mechanisms of Astragalus-derived compounds against age-related hearing loss (ARHL), a network pharmacology approach was employed. The chemical structures of Astragaloside IV (AS-IV) and Cycloastragenol (CAG) were retrieved from the PubChem database. Putative target genes for both AS-IV and CAG were independently predicted using standard pharmacological databases (SwissTargetPrediction). The respective targets of AS-IV and CAG were subsequently pooled to obtain the union dataset, representing the comprehensive pharmacological target profile of the active compounds.

Concurrently, disease-associated targets were gathered by searching two major human genetic databases, GeneCards and OMIM (Online Mendelian Inheritance in Man). Various keywords related to the pathology, including “Age-Related Hearing Loss,” “Sensorineural Hearing Loss,” and “Cellular Senescence,” were utilized. The search results were consolidated, and duplicate genes were removed to establish a comprehensive database of ARHL-related targets.

To identify the core therapeutic targets, the union dataset of the compound targets (AS-IV and CAG) was mapped against the ARHL-related disease targets. The overlapping genes between the compounds and the disease were extracted as the potential therapeutic targets of AS-IV and CAG against ARHL. These overlapping targets were then imported into the STRING database to construct a Protein–Protein Interaction (PPI) network [39]. The PPI network data was visualized and analyzed using Cytoscape software (Version 3.8.2) [40]. Topological analysis was performed using the NetworkAnalyzer plugin, and the top 20 core targets were screened and selected based on key topological parameters, primarily the degree centrality.

To explore the biological functions and signaling pathways associated with the overlapping targets, Gene Ontology (GO) annotation and Kyoto Encyclopedia of Genes and Genomes (KEGG) pathway enrichment analyses were conducted using the DAVID or Metascape bioinformatics databases. Statistical significance was determined using a threshold of *p* < 0.05. The highly enriched pathways, particularly those related to apoptosis, longevity, and mitochondrial energy metabolism, were selected for graphical visualization.

To explore computationally plausible ligand–target poses among network-associated proteins, molecular docking was performed using AutoDock Vina (Version 1.2.0) [41]. The 3D structures of AS-IV and CAG were energy-minimized and prepared as ligands using AutoDockTools. Crystal structures of the 20 selected target proteins were obtained from the RCSB Protein Data Bank (PDB). Receptor structures were prepared by removing co-crystallized water molecules and native ligands, adding polar hydrogens, and assigning Gasteiger charges. Target-specific grid boxes (28 × 28 × 28 Å) were centered on the selected binding sites. Docking was performed with an exhaustiveness of 8, 10 output modes, an energy range of 4 kcal/mol, and a fixed random seed of 20260713; for the highly flexible AS-IV ligand, the maximum number of evaluations was set to 50,000. AutoDock Vina scores were used to rank candidate poses and were not interpreted as experimentally measured binding affinities or free energies. The lowest-score CAG poses selected for visualization were analyzed using PyMOL (Version 3.0, Schrödinger, Inc., New York, NY, USA) and Discovery Studio Visualizer (Version v21.1.0.20298, Dassault Systèmes, San Diego, CA, USA).

### 2.11. Statistical Analysis and Generative AI Declaration

Statistical analyses were performed using GraphPad Prism 10.0 (GraphPad Software, San Diego, CA, USA). Data are presented as mean ± standard deviation (SD). Multiple-group comparisons were performed using one-way analysis of variance (ANOVA), followed by Tukey’s honestly significant difference (HSD) test for pairwise multiple comparisons when the omnibus test was significant. ABR thresholds were analyzed separately at each stimulus frequency using one-way ANOVA; frequency was not included as a repeated-measures factor. A *p*-value of <0.05 was considered statistically significant (* *p* < 0.05, ** *p* < 0.01, *** *p* < 0.001).

Generative artificial intelligence tools (ChatGPT, OpenAI, version GPT 5.6; DeepSeek, DeepSeek AI, version DeepSeek-V4) were used to assist the authors during manuscript preparation. Specifically, these tools were employed to (i) retrieve and organize relevant literature from the local reference database (Zotero); (ii) summarize and cross-check bibliographic information; and (iii) improve the clarity and language of the manuscript text. No AI-generated text, data, figures, or study design elements were incorporated into this work without thorough manual verification: all outputs were checked against the original sources and revised by the authors. The authors confirm that the study design, data collection, analysis, and interpretation were performed by the authors themselves, and they assume full responsibility for the accuracy and integrity of the reported results.

## 3. Results

### 3.1. Cycloastragenol and Astragaloside IV Ameliorate D-Galactose-Induced Cellular Senescence and Oxidative Stress in HEI-OC1 Cells

#### 3.1.1. Establishment and Validation of the D-Galactose-Induced Senescence Model

**Figure 1:**
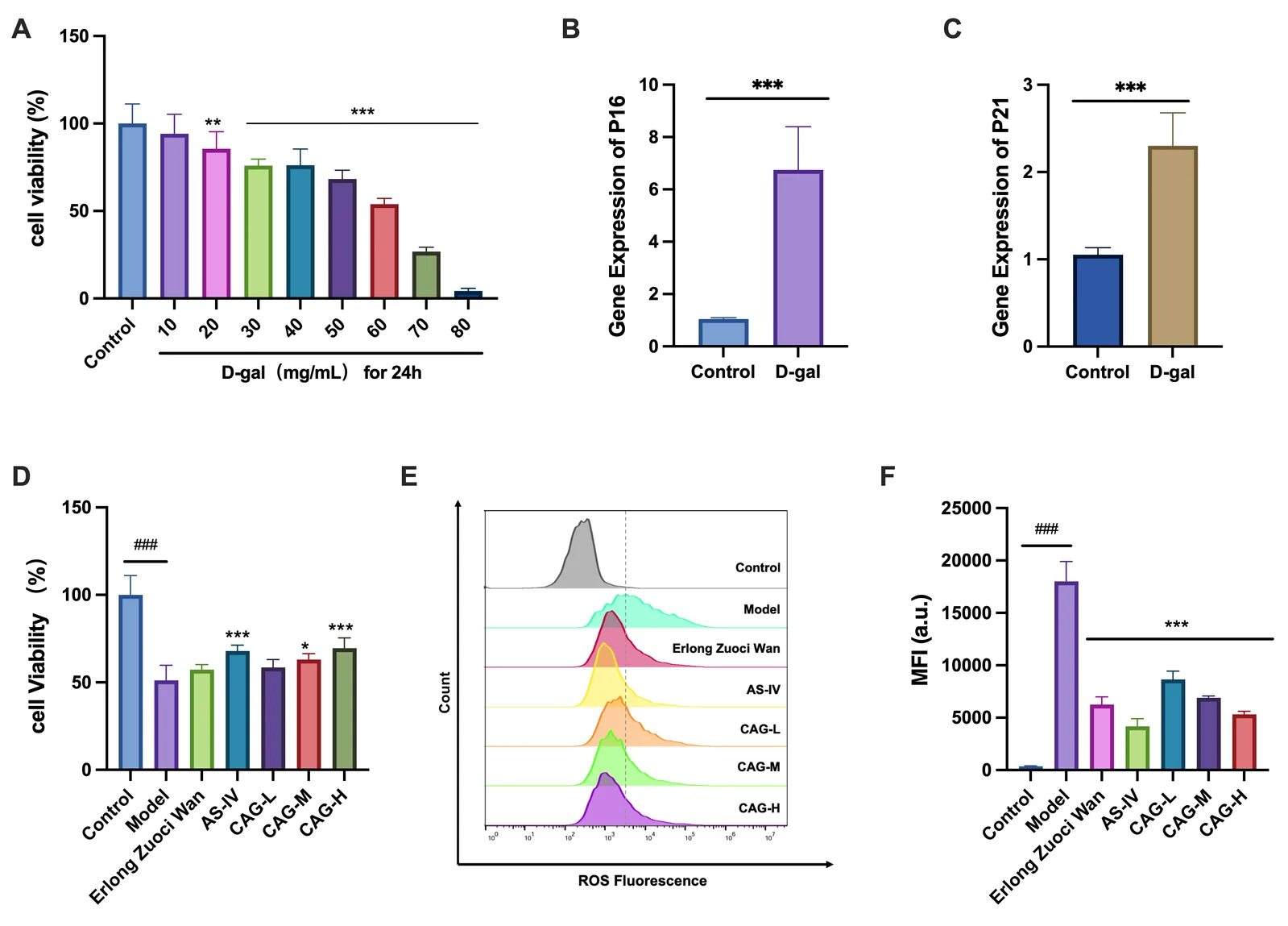
Establishment of D-galactose-induced senescence model in HEI-OC1 cells and the in vitro protective effects of Astragaloside IV and Cycloastragenol. (**A**) Cell viability of HEI-OC1 cells treated with various concentrations of D-gal (0–80 mg/mL) for 24 h, measured by CCK-8 assay, identifying 40 mg/mL as the optimal modeling concentration. (**B**,**C**) Relative mRNA expression levels of senescence markers P16 (**B**) and P21 (**C**) in HEI-OC1 cells after exposure to 40 mg/mL D-gal for 24 h, analyzed by RT-qPCR. (**D**) Protective effects of Astragaloside IV (50 μM), Cycloastragenol (10, 25, 50 μM), and Erlong Zuoci Wan on the viability of D-gal-induced senescent HEI-OC1 cells. (**E**,**F**) Flow-cytometric analysis of intracellular ROS in the D-gal-induced model. (**E**) Representative ROS fluorescence histograms; leftward shifts indicate lower DCFH-DA fluorescence. (**F**) Quantitative mean fluorescence intensity (MFI) analysis. Erlong Zuoci Wan, AS-IV, and CAG reduced ROS MFI relative to the Model group; CAG showed a concentration-related effect across the tested concentrations. Data are presented as mean ± SD. For cell viability and ROS assays, n = 6; for RT-qPCR assays, n = 3. * *p* < 0.05, ** *p* < 0.01, and *** *p* < 0.001 versus the indicated comparison group; ### *p* < 0.001 versus the Model group.

To establish a stable in vitro model of age-related hair cell degeneration, HEI-OC1 cells were exposed to varying concentrations of D-galactose (D-gal) (0–80 mg/mL) for 24 h. As evaluated by the CCK-8 assay, D-gal treatment induced a dose-dependent decrease in cell viability. Significant cytotoxicity was observed starting at 20 mg/mL (*p* < 0.01), with viability sharply declining at higher doses. To induce robust cellular senescence while avoiding excessive acute cell death, 40 mg/mL of D-gal for 24 h was selected as the optimal modeling condition for subsequent experiments (Figure 1A).

To confirm that the viability loss was driven by cellular senescence at the molecular level, the expression of classical senescence marker genes, P16 and P21, was measured using RT-qPCR. P21, a cyclin-dependent kinase inhibitor regulated by the p53 pathway, acts as an early marker for DNA damage and oxidative stress, while P16 serves as a stable marker of irreversible cell cycle arrest. Following exposure to 40 mg/mL D-gal, the mRNA expression levels of both P16 and P21 were highly significantly upregulated compared to the control group (*p* < 0.001) (Figure 1B,C). The synchronous elevation of these markers confirmed the successful establishment of the HEI-OC1 cellular senescence model.

#### 3.1.2. CAG and AS-IV Restore Cell Viability in Senescent HEI-OC1 Cells

Using the established model, the protective efficacies of the Astragalus-derived compounds, Cycloastragenol (CAG) and its precursor Astragaloside IV (AS-IV), were evaluated. The traditional Chinese medicine Erlong Zuoci Wan was utilized as a positive control. The results demonstrated that co-treatment with AS-IV (50 μM) and CAG (50 μM) significantly rescued the D-gal-induced decline in cell viability (*p* < 0.001). Notably, CAG exhibited a clear dose-dependent protective effect across 10, 25, and 50 μM concentrations. Furthermore, both CAG and AS-IV demonstrated superior efficacy in restoring cell viability compared to the traditional clinical formulation control (Figure 1D).

#### 3.1.3. CAG and AS-IV Suppress D-Gal-Induced Intracellular ROS Accumulation

Intracellular ROS was quantified by DCFH-DA flow cytometry. Compared with the Control group, D-gal exposure significantly increased ROS mean fluorescence intensity (MFI), indicating intracellular accumulation of oxidant-sensitive fluorescence. Erlong Zuoci Wan, AS-IV (50 μM), and CAG (10, 25, and 50 μM) each shifted the ROS fluorescence distribution leftward and reduced MFI relative to the Model group (*p* < 0.001; Figure 1E,F). The greatest leftward shift was observed in the AS-IV group, whereas the CAG groups showed a concentration-related reduction in MFI. These results support the antioxidant activity of AS-IV and CAG in the D-gal-induced HEI-OC1 model.

### 3.2. Cycloastragenol and Astragaloside IV Stabilize Mitochondrial Membrane Potential and Attenuate D-Galactose-Induced Apoptosis in HEI-OC1 Cells

#### 3.2.1. AS-IV and CAG Effectively Reverse D-Gal-Induced Mitochondrial Membrane Potential Collapse

Mitochondria are the primary source of intracellular ROS and the principal targets of oxidative damage. The mitochondrial membrane potential (MMP), generated by the proton gradient across the electron transport chain, is the fundamental driving force for ATP synthesis. In the D-gal-induced senescence model, excessive ROS accumulation attacks the respiratory chain complexes on the inner mitochondrial membrane, leading to decreased proton pump efficiency and structural impairment. This triggers an irreversible collapse of the MMP, which serves as a hallmark event in early cellular apoptosis.

**Figure 2:**
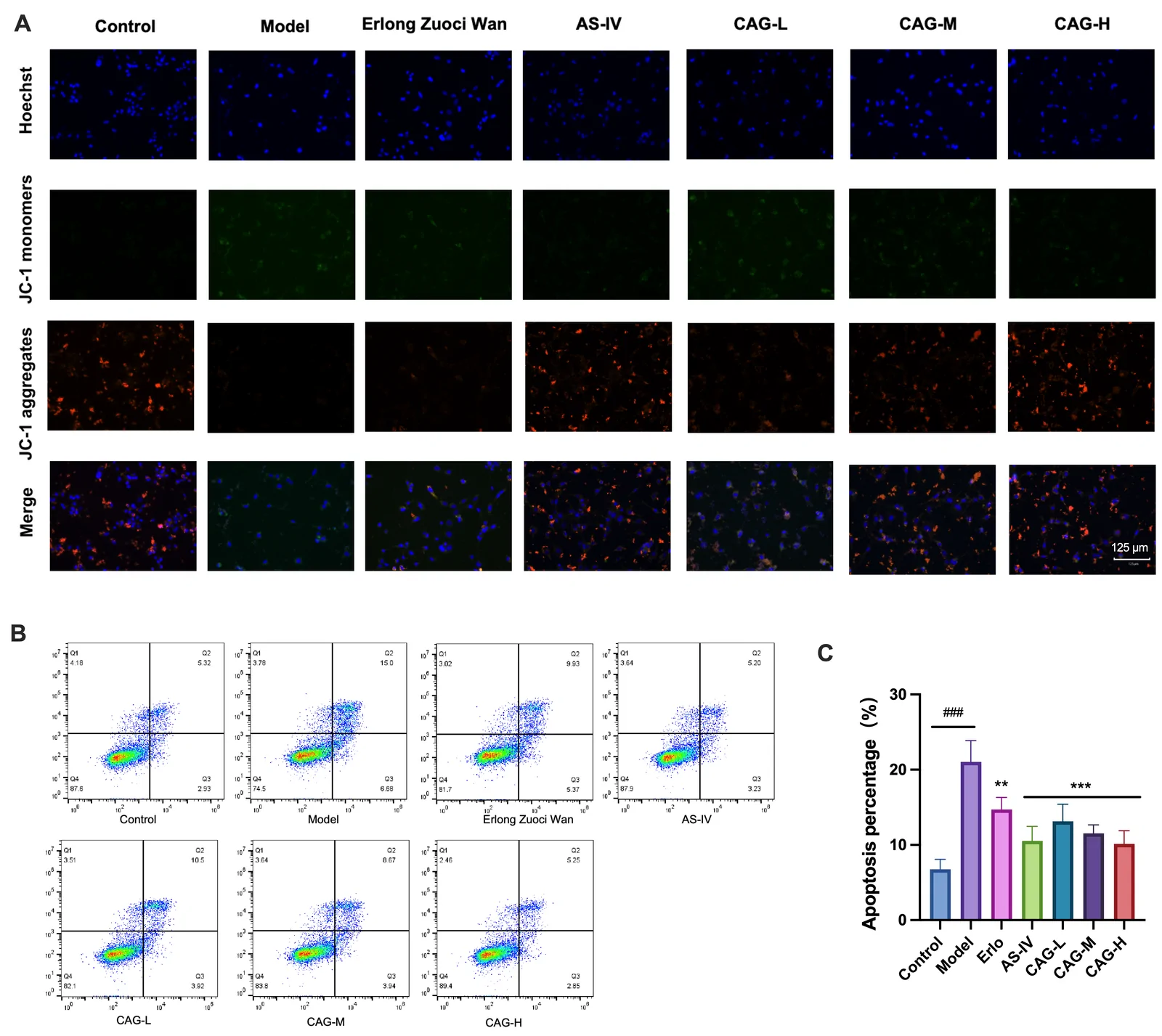
Astragaloside IV and Cycloastragenol stabilize mitochondrial membrane potential and alleviate cell apoptosis in D-galactose-induced HEI-OC1 cells. (**A**) Representative fluorescence micrographs of JC-1 staining demonstrating changes in mitochondrial membrane potential (MMP). Blue fluorescence indicates Hoechst 33,342-stained nuclei; green fluorescence represents JC-1 monomers (low MMP); red fluorescence represents J-aggregates (high MMP); “Merge” displays the superimposed channels. Scale bar = 125 μm. Images were captured at ×200 magnification. **(**B) Representative flow cytometry quadrant plots of Annexin V-FITC/PI double staining showing the distribution of apoptotic HEI-OC1 cells across different groups. (**C**) Quantitative analysis of the total apoptosis percentage (comprising both early and late apoptotic cells). Data are presented as mean ± SD (n = 6). ### *p* < 0.001 versus the Control group; ** *p* < 0.01, and *** *p* < 0.001 versus the Model group, as indicated in the panels.

To evaluate the protective effects of the compounds on mitochondrial structural integrity, MMP was assessed using the JC-1 fluorescent probe. Fluorescence microscopy revealed that in the untreated Control group, JC-1 primarily formed J-aggregates within the mitochondrial matrix, exhibiting intense red fluorescence (Figure 2A). Conversely, HEI-OC1 cells in the D-gal-induced Model group displayed a pronounced reduction in red fluorescence and a concomitant widespread increase in green monomer fluorescence, indicating severe MMP depolarization and mitochondrial dysfunction. Treatment with the Astragalus-derived compounds provided evident rescue effects. AS-IV (50 μM) effectively preserved the red fluorescence and minimized the green signal, signifying robust stabilization of the MMP. Furthermore, CAG (10, 25, and 50 μM) exerted a clear, dose-dependent protective effect; as the concentration of CAG increased, the intensity of the red J-aggregates progressively recovered while the green monomer fluorescence continuously diminished. Notably, with the exception of the low-dose CAG group, the green fluorescence intensity in the AS-IV and the mid-to-high dose CAG groups was visibly lower than that of the clinical positive control (Erlong Zuoci Wan). These results suggest that these active monomers possess superior targeted efficacy in maintaining mitochondrial homeostasis and preventing the early stages of cellular apoptosis.

#### 3.2.2. AS-IV and CAG Significantly Block the D-Gal-Activated Intrinsic Apoptotic Program

The severe oxidative stress and mitochondrial impairment associated with cellular senescence inevitably trigger intrinsic apoptotic cascades. When the protective “growth arrest” mechanisms of senescence are overwhelmed by excessive damage, cells transition towards programmed cell death. By scavenging ROS and preserving mitochondrial functional integrity, pharmacological interventions can fundamentally block this mitochondria-dependent apoptosis pathway at its source.

To quantify this protection, cellular apoptosis was evaluated using Annexin V-FITC/PI double staining combined with flow cytometry (Figure 2B,C). The Annexin V-positive/PI-negative population was defined as early apoptotic cells, while the double-positive population represented late apoptotic or necrotic cells. D-gal exposure increased the total apoptosis rate in the Model group compared with the baseline. Interventions with AS-IV (50 μM) and CAG (10, 25, and 50 μM) significantly reduced the total apoptotic rates compared with the Model group (*p* < 0.001). Consistent with the MMP findings, both AS-IV and CAG showed stronger anti-apoptotic effects than Erlong Zuoci Wan under these in vitro conditions. These quantitative flow-cytometry data support an association between AS-IV or CAG treatment and reduced D-gal-induced HEI-OC1 cell apoptosis.

### 3.3. CAG and AS-IV Suppress the Intrinsic Mitochondrial Apoptosis Pathway by Regulating the Bax/Bcl-2 Balance and Caspase-3 Activation

**Figure 3:**
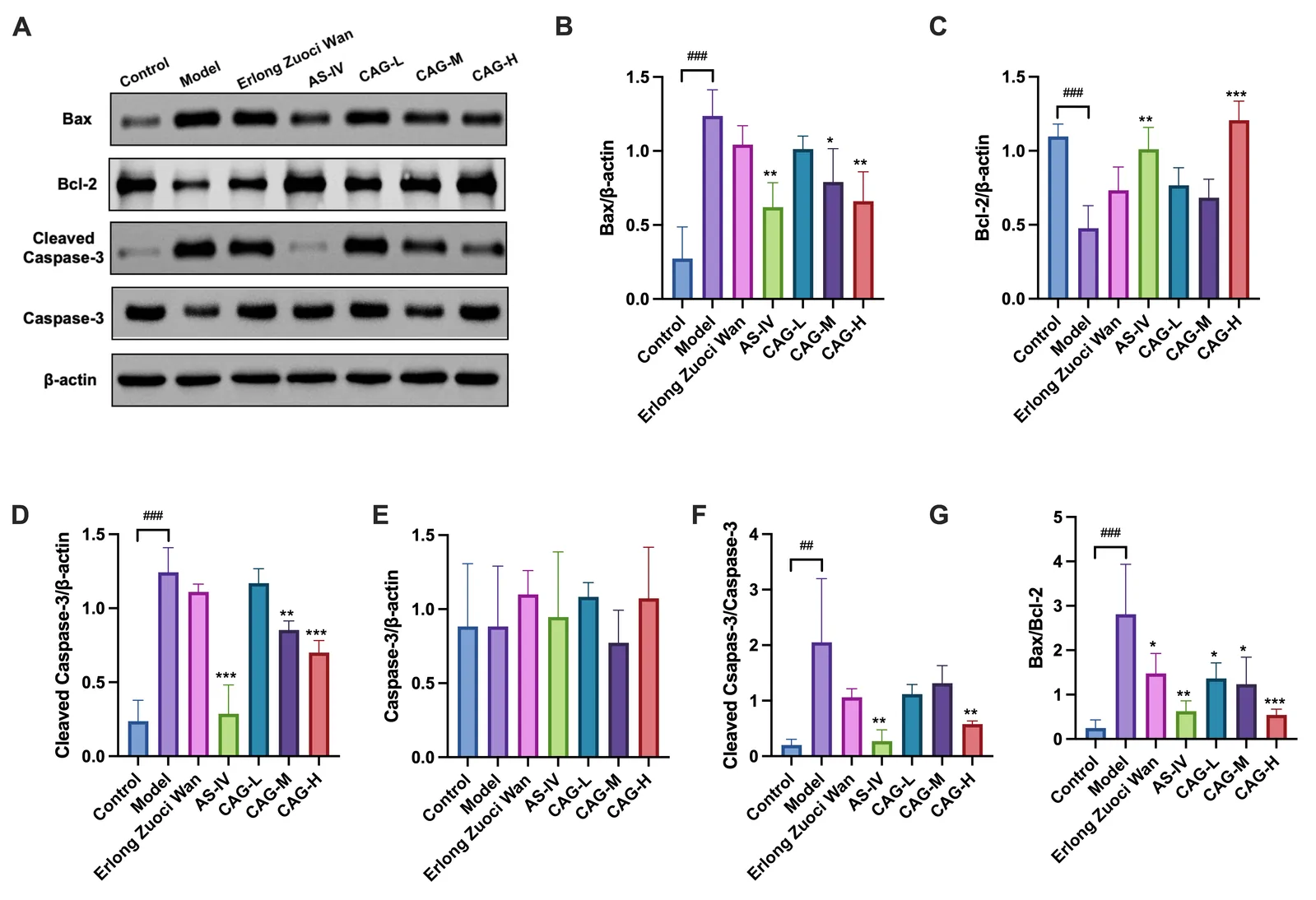
Astragaloside IV and Cycloastragenol inhibit D-galactose-induced HEI-OC1 cell apoptosis by regulating the Bax/Bcl-2/Caspase-3 signaling pathway. (**A**) Representative Western blot images showing the protein expression levels of Bax, Bcl-2, Cleaved Caspase-3, and total Caspase-3 in HEI-OC1 cells across different treatment groups. (**B**–**D**) Quantitative statistical analysis of the relative protein expression of Bax (**B**), Bcl-2 (**C**), and the pro-apoptotic to anti-apoptotic Bax/Bcl-2 ratio (**D**). (**E**–**G**) Quantitative statistical analysis of the relative protein expression of Cleaved Caspase-3 (**E**), total Caspase-3 (**F**), and the ratio of Cleaved Caspase-3 to total Caspase-3 (**G**), reflecting the activation of the executioner caspase. Data are presented as mean ± SD (n = 6). ## *p* < 0.01 and ### *p* < 0.001 versus the Control group; * *p* < 0.05, ** *p* < 0.01, and *** *p* < 0.001 versus the Model group.

The mitochondrial apoptotic cascade represents a core regulatory pathway governing cellular senescence. Within this pathway, the ratio of the pro-apoptotic protein Bax to the anti-apoptotic protein Bcl-2 acts as a molecular rheostat that governs mitochondrial outer membrane permeability. An elevated Bax/Bcl-2 ratio precipitates the release of mitochondrial cytochrome c into the cytosol, which subsequently triggers the downstream caspase cascade and activates Caspase-3 to execute programmed cell death, ultimately accelerating the senescence process. To elucidate the molecular mechanisms underlying the protective effects of Astragaloside IV (AS-IV) and Cycloastragenol (CAG), the expression levels of these key apoptosis-related proteins in HEI-OC1 cells were quantified via Western blot analysis (Figure 3A).

Compared with the untreated Control group, D-gal exposure in the Model group significantly increased Bax and cleaved caspase-3 expression, leading to higher Bax/Bcl-2 and cleaved caspase-3/caspase-3 ratios (### *p* < 0.001, ## *p* < 0.01; Figure 3B,D,E,G). Concurrently, the expression of the anti-apoptotic protein Bcl-2 was reduced in the Model group (### *p* < 0.001; Figure 3C). These findings indicate that D-gal exposure was associated with activation markers of the intrinsic mitochondrial apoptosis pathway in HEI-OC1 cells.

Intervention with either AS-IV or varying concentrations of CAG countered these apoptosis-related alterations. Compared with the Model group, co-treatment with AS-IV (50 μM) or CAG (10, 25, and 50 μM) reduced the Bax/Bcl-2 and cleaved caspase-3/caspase-3 ratios to varying degrees (Figure 3D,G). The 50 μM CAG group showed the most pronounced reduction among the CAG-treated groups and was comparable to the AS-IV group. In contrast, total caspase-3 expression remained statistically unaltered across all experimental groups (Figure 3F), suggesting that the observed changes primarily reflected altered caspase-3 cleavage rather than total caspase-3 abundance. Taken together, these data indicate that AS-IV and CAG were associated with normalization of the Bax/Bcl-2 balance and reduced caspase-3 activation markers in D-gal-induced HEI-OC1 cells.

### 3.4. CAG Attenuated Auditory Dysfunction and Hair Cell Loss in D-Galactose-Treated Rats

#### 3.4.1. CAG Reduced ABR Thresholds in D-Galactose-Treated Rats

Auditory function was evaluated by ABR at baseline and at the study endpoint following 8 weeks of D-galactose modeling and 4 weeks of treatment. Thresholds were measured at 4, 8, 16, and 32 kHz in the Control, Model, GP, CAG-L, and CAG-H groups.

**Figure 4:**
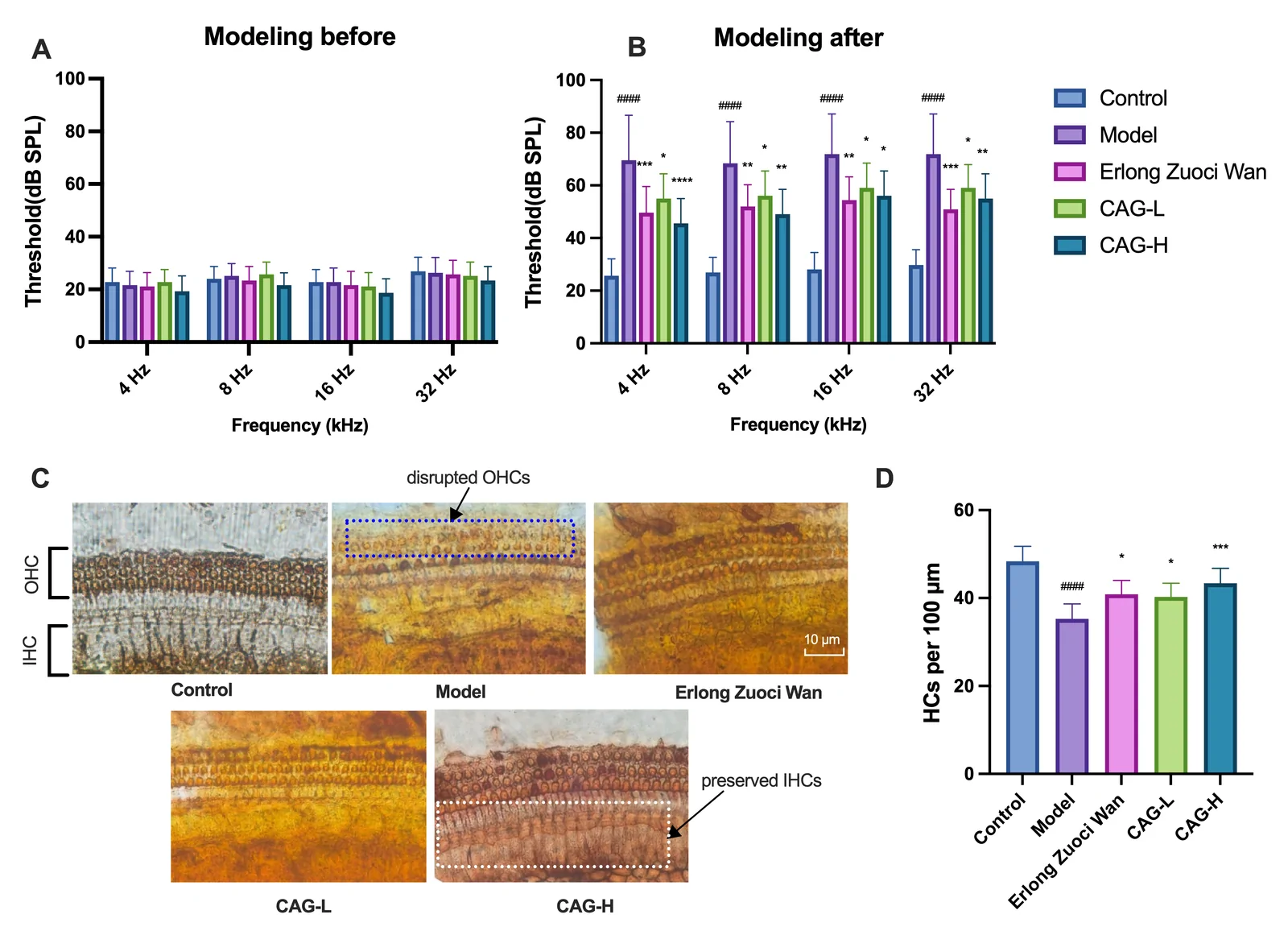
Cycloastragenol (CAG) attenuates auditory dysfunction and hair cell loss in D-galactose-treated rats. (**A**,**B**) Auditory brainstem response (ABR) thresholds at 4, 8, 16, and 32 kHz at baseline (**A**) and at the study endpoint following 8 weeks of D-galactose modeling and 4 weeks of treatment (**B**). The Model group received D-galactose (1000 mg/kg/day, i.p.) during modeling; GP, CAG-L, and CAG-H received Erlong Zuoci Wan (100 mg/kg/day), CAG (5 mg/kg/day), or CAG (20 mg/kg/day), respectively, by oral gavage during the 4-week treatment period. (**C**) Representative micrographs of silver nitrate (AgNO_3_) staining of the cochlear basilar membrane surface preparations, illustrating the structural arrangement and preservation of cochlear hair cells across the experimental groups. IHC, inner hair cells; OHC, outer hair cells. In the Model panel, the blue dashed box highlights the disrupted OHC region, in which the number of the three OHC rows was reduced and their morphology appeared shrunken. In the CAG-H panel, the white dashed box highlights the preserved IHCs, which exhibited a regular cell arrangement. Scale bar = 10 μm. Images were captured at ×400 magnification. (**D**) Number of surviving hair cells per 100 μm of cochlear surface preparation in each group. Experimental groups (n = 6 rats per group): Control (0.5% CMC-Na), Model (D-gal plus 0.5% CMC-Na), GP (Erlong Zuoci Wan, 100 mg/kg/day), CAG-L (5 mg/kg/day), and CAG-H (20 mg/kg/day). Data are presented as mean ± SD. #### *p* < 0.0001 versus the Control group; * *p* < 0.05, ** *p* < 0.01, *** *p* < 0.001, and **** *p* < 0.0001 versus the Model group.

At the study endpoint following 8 weeks of D-galactose modeling and 4 weeks of treatment, ABR thresholds in the D-gal Model group were higher than those in the Control group at all tested frequencies (*p* < 0.0001; Figure 4A,B). CAG treatment at 5 and 20 mg/kg was associated with lower thresholds than the Model group across the tested frequencies. At selected frequencies, thresholds also differed between the CAG-H and CAG-L groups (*p* < 0.05). These findings indicate that CAG attenuated the auditory threshold elevations associated with chronic D-galactose administration followed by vehicle treatment.

#### 3.4.2. CAG Preserved Cochlear Hair Cell Architecture

Cochlear surface preparations were stained with silver nitrate to assess hair-cell organization and survival at the study endpoint. Silver nitrate staining showed an ordered hair cell arrangement in the Control group, whereas the D-gal Model group displayed disrupted organization and hair cell loss (Figure 4C). Both CAG-treated groups showed greater preservation of hair cell architecture than the Model group, with the CAG-H group exhibiting the most continuous arrangement among the treated groups.

Hair cells were quantified per 100 μm of cochlear surface preparation (Figure 4D). The Model group had fewer surviving hair cells than the Control group, whereas both CAG-treated groups retained more hair cells than the Model group. These morphological findings were consistent with the ABR results and support an association between CAG treatment and reduced cochlear injury in D-galactose-treated rats.

### 3.5. CAG Was Associated with Improved Cochlear Redox Indices and Lower Serum Cytokine Levels

#### 3.5.1. CAG Improved Cochlear Redox-Related Indices

To characterize redox-related changes associated with CAG treatment, SOD, GSH, MDA, and ROS were quantified in cochlear homogenates collected at the study endpoint.

**Figure 5:**
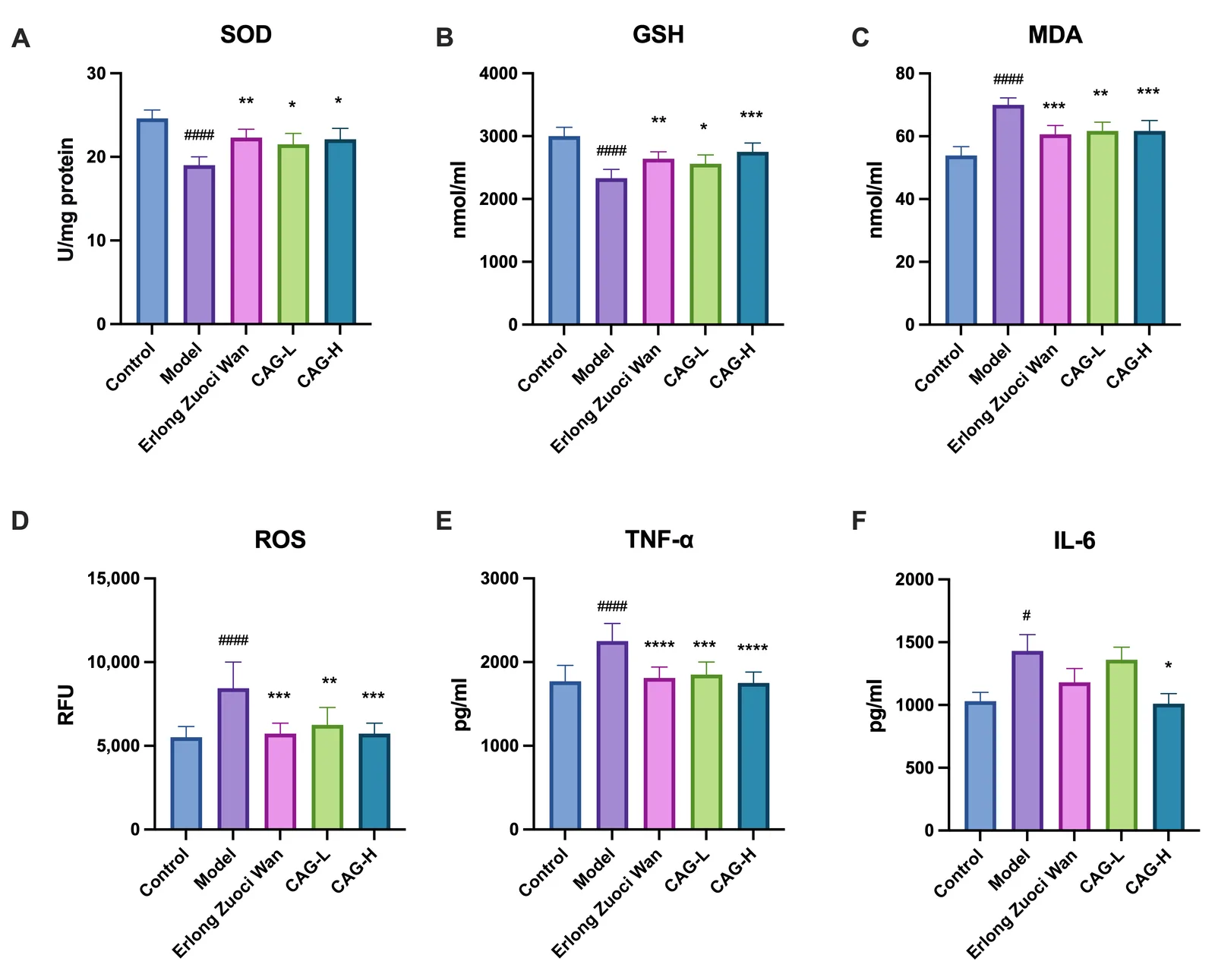
Cycloastragenol (CAG) was associated with improved cochlear redox indices and lower serum cytokine levels in D-galactose-treated rats. (**A**,**B**) Cochlear superoxide dismutase (SOD) and glutathione (GSH) levels. (**C**,**D**) Cochlear malondialdehyde (MDA) and reactive oxygen species (ROS) levels. (**E**,**F**) Serum tumor necrosis factor-α (TNF-α) and interleukin-6 (IL-6) concentrations. Data are presented as mean ± SD (n = 6 rats per group). Groups: Control, Model (D-gal, 1000 mg/kg/day), GP (Erlong Zuoci Wan, 100 mg/kg/day), CAG-L (5 mg/kg/day), and CAG-H (20 mg/kg/day). Statistical symbols denote comparisons with the Control or Model group as indicated in the panels. # *p* < 0.05 and #### *p* < 0.0001 versus the Control group; * *p* < 0.05, ** *p* < 0.01, *** *p* < 0.001, and **** *p* < 0.0001 versus the Model group.

Compared with the Control group, the D-gal Model group showed lower cochlear SOD and GSH levels and higher MDA and ROS levels (Figure 5A–D). CAG-H treatment (20 mg/kg) was associated with higher SOD and GSH and lower MDA and ROS than the Model group. These changes were directionally consistent with the cellular ROS findings and indicate that CAG treatment was associated with improved cochlear redox status under the conditions tested.

#### 3.5.2. CAG Reduced Serum TNF-α and IL-6 Levels

To assess systemic inflammatory changes associated with the intervention, TNF-α and IL-6 concentrations were measured in serum collected at the study endpoint after the 4-week treatment period.

Serum TNF-α and IL-6 concentrations were higher in the D-gal Model group than in the Control group (Figure 5E,F). CAG-H treatment was associated with lower concentrations of both cytokines than the Model group. Because inflammatory mediators were measured in serum rather than cochlear tissue, these findings indicate a change in systemic inflammatory status and do not establish a direct local anti-inflammatory effect in the cochlea.

### 3.6. Comparative Pharmacokinetics, Cochlear Exposure, and Microsomal Biotransformation of CAG and AS-IV

#### 3.6.1. Direct CAG Administration Produced Greater Systemic CAG Exposure than AS-IV Administration

To elucidate the translational advantages of utilizing the aglycone (CAG) over its macromolecular precursor (AS-IV), a comprehensive in vivo pharmacokinetic study was conducted in C57BL/6 mice. Following a single oral administration of 20 mg/kg, a validated LC-MS/MS method was employed to simultaneously monitor the concentrations of both CAG and AS-IV in the biological matrices.

**Figure 6:**
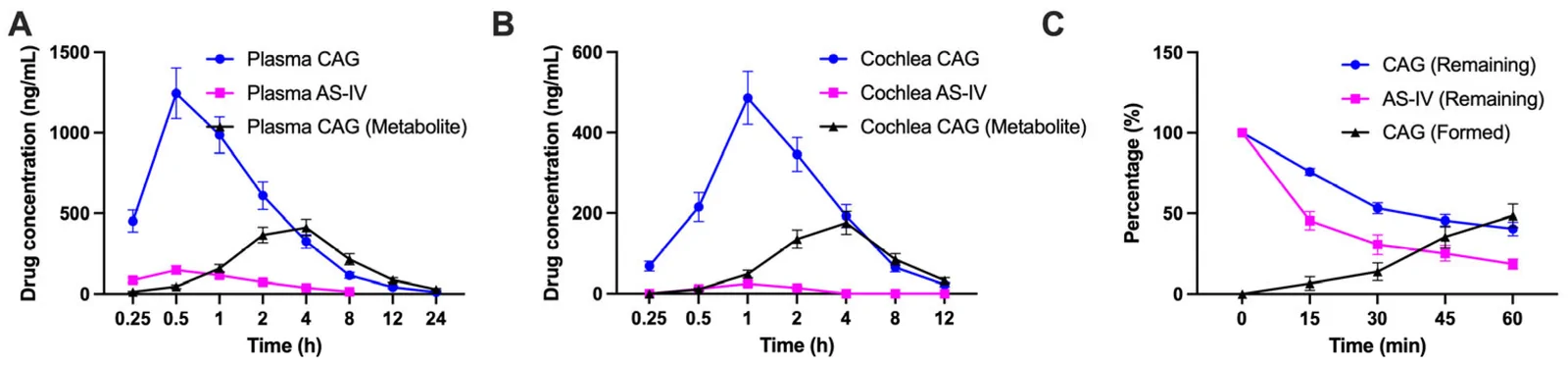
Comparative plasma pharmacokinetics, exposure in perfused whole cochleae, and mouse liver microsomal biotransformation of CAG and AS-IV. (**A**) Mean plasma concentration-time profiles after a single oral dose (20 mg/kg) in male C57BL/6 mice. Directly administered CAG showed rapid absorption and greater systemic exposure (Blue). In the AS-IV-administered group, both AS-IV (Magenta) and its metabolite CAG (Black) were simultaneously detected, illustrating the gradual in vivo biotransformation of the saponin into its aglycone. (**B**) Cochlear tissue concentration-time curves demonstrating that direct administration of CAG (Blue) produced higher CAG exposure in perfused whole-cochlea homogenates than CAG formed after AS-IV administration; these data do not directly measure BLB permeability. Blue represents CAG in the CAG-administered group, while Magenta and black represent AS-IV and CAG, respectively in AS-IV administered group. (**C**) Ex vivo metabolic stability in mouse liver microsomes. The curves depict the time-dependent loss of AS-IV (magenta) accompanied by the formation of CAG (black). At 60 min, AS-IV remaining decreased to 18.5% ± 3.2%, CAG formed from AS-IV increased to 48.6% ± 7.2%, and directly incubated CAG showed 40.4% ± 4.2% remaining, supporting microsomal AS-IV-to-CAG biotransformation and indicating that CAG also underwent microsomal loss under the assay conditions. Data are presented as mean ± SD (n = 6 mice per time point for the in vivo studies).

The plasma concentration-time profiles revealed stark disparities in systemic absorption and metabolism. In the CAG-administered group, CAG was rapidly absorbed into the systemic circulation, achieving a high maximum plasma concentration (C_max_) of 1285.4 ± 162.5 ng/mL at a rapid T_max_ of 0.5 h, and maintaining robust overall exposure (AUC_0–24h_ = 5642.8 ± 485.2 h·ng/mL) (Figure 6A, Table 1).

Strikingly, in the AS-IV-administered group, systemic exposure to the parent saponin was exceedingly poor (C_max_ = 155.2 ± 28.4 ng/mL). However, both AS-IV and its aglycone metabolite (CAG) were simultaneously detected in the plasma (Figure 6A). Over the 24 h monitoring period, the concentration of the parent AS-IV gradually declined, accompanied by a corresponding emergence of CAG, which peaked much later (T_max_ = 4.0 h) at 425.6 ± 55.8 ng/mL. This distinct pharmacokinetic phenomenon provides direct in vivo evidence that orally administered AS-IV can serve as a metabolic precursor of CAG under the tested conditions. The total plasma exposure of CAG after AS-IV administration (AUC_0–24h_ = 3125.4 ± 350.6 h·ng/mL) was nearly half of that achieved by direct CAG administration.

#### 3.6.2. Direct CAG Administration Increased Exposure in Perfused Whole Cochleae

Analysis of transcardially perfused whole cochleae showed measurable CAG after direct oral administration, with a cochlear C_max_ = 495.6 ± 72.1 ng/g) (Figure 6B). The cochlea-to-plasma partition coefficient (K_p_) was 0.35 ± 0.04. These measurements demonstrate cochlear exposure but do not directly establish transport across the intact blood-labyrinth barrier.

In contrast, the concentration of intact AS-IV in perfused whole-cochlea homogenates was lower (C_max_ = 26.8 ± 7.5 ng/g, K_p_ = 0.09 ± 0.02). CAG formed after AS-IV administration was also detected in the cochlear homogenates (K_p_ = 0.33 ± 0.05); its peak concentration in the target site (C_max_ = 182.5 ± 31.2 ng/g) was lower than that observed after direct CAG administration (Table 1). Thus, direct CAG administration produced greater cochlear CAG exposure under the tested dosing conditions. These tissue-distribution data do not identify the route of entry into the cochlea or directly measure BLB permeability.

#### 3.6.3. Mouse Liver Microsomes Supported AS-IV-to-CAG Biotransformation Under the Tested Conditions

To evaluate whether mouse liver microsomes could support the observed in vivo AS-IV-to-CAG biotransformation, the metabolic stability of the compounds was investigated using an ex vivo mouse liver microsomal incubation assay (Figure 6C).

In the newly selected microsomal time-course dataset, AS-IV concentration declined over the incubation period while CAG formation increased (Figure 6C). At 60 min, AS-IV remaining decreased to 18.5 ± 3.2%, while CAG formation increased to 48.6 ± 7.2%. Directly incubated CAG also showed time-dependent loss, with 40.4 ± 4.2% remaining at 60 min (Figure 6C). Together, these observations support the capacity of mouse liver microsomes to convert AS-IV to CAG and suggest that the biotransformation observed in vivo may include hepatic enzymatic deglycosylation. Because liver microsomes model metabolic capacity rather than the anatomical contribution of each organ in vivo, these data do not exclude gastrointestinal conversion of AS-IV. Direct CAG administration reduced reliance on AS-IV-to-CAG conversion but did not eliminate CAG first-pass metabolism.

### 3.7. Systems Pharmacology Nominated SIRT1-Related and Apoptosis-Associated Pathways as Candidate Mechanisms

#### 3.7.1. Target Identification and PPI Network Construction

**Figure 7:**
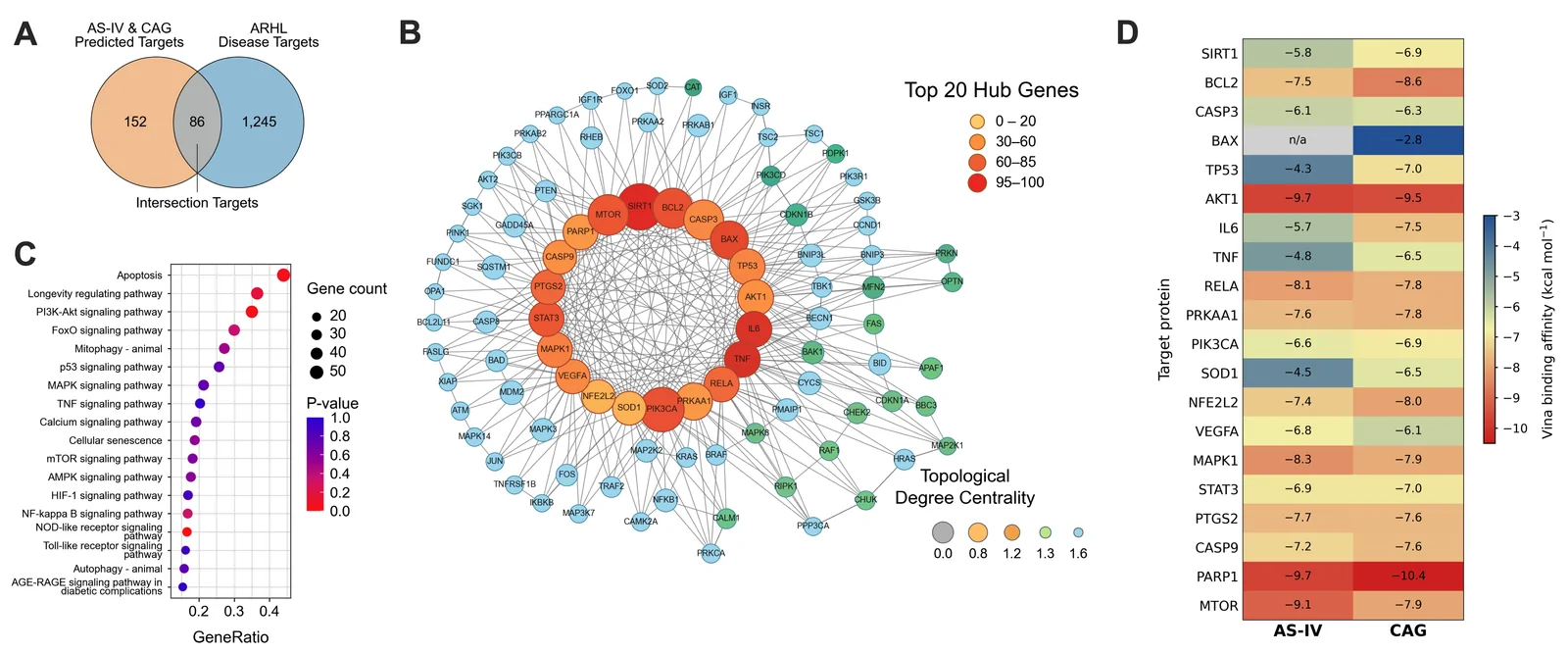
Systems-pharmacology and molecular-docking analyses nominated candidate pathways and protein interactions associated with AS-IV and CAG. (**A**) Intersection of predicted compound targets with genes associated with age-related hearing loss and cellular senescence. (**B**) Protein–protein interaction network of the shared candidate targets; the 20 nodes with the highest degree centrality are highlighted. (**C**) KEGG enrichment analysis of the shared candidate targets, including apoptosis, longevity-regulating, and PI3K-Akt signaling pathways. Enrichment denotes database over-representation and does not demonstrate pathway activation. (**D**) Heatmap of AutoDock Vina scores (kcal/mol) for AS-IV and CAG across the 20 selected target proteins. Scores are computational ranking estimates rather than experimentally measured binding affinities. The positive AS-IV–BAX score was considered uninterpretable and is shown as n/a.

To generate testable hypotheses regarding potential molecular associations of AS-IV and CAG, we performed a systems-pharmacology analysis. Reverse pharmacophore mapping and public databases yielded 152 non-redundant putative compound targets, whereas GeneCards and OMIM yielded 1245 genes associated with age-related hearing loss and cellular senescence. Intersection of these datasets identified 86 shared candidate targets (Figure 7A).

To determine the network connectivity of the shared targets, a protein–protein interaction network was constructed using STRING and visualized in Cytoscape. Degree centrality identified 20 highly connected nodes, including SIRT1 and PRKAA1, apoptosis-associated proteins (BCL2, CASP3, BAX, and TP53), and inflammatory mediators (TNF and IL6) (Figure 7B). Their occurrence was consistent with the redox- and apoptosis-related phenotypes observed experimentally but did not establish that these proteins were directly modulated by CAG.

#### 3.7.2. Functional Enrichment Highlights Apoptosis and Longevity Pathways

KEGG enrichment analysis of the 86 shared candidate targets identified apoptosis, longevity-regulating, and PI3K-Akt signaling among the prominently represented pathways (Figure 7C). These associations provided a hypothesis-generating framework linking the computational target set with the observed redox and apoptosis phenotypes. Because enrichment reflects database overlap rather than pathway activity, these analyses do not demonstrate pathway regulation by either compound.

#### 3.7.3. Molecular Docking-Generated Candidate CAG-Protein Interaction Poses

AutoDock Vina was used to evaluate computationally plausible binding poses of AS-IV and CAG with selected network-associated proteins. The resulting docking scores are model-dependent estimates used to prioritize candidate interactions and should not be interpreted as measured binding free energies or evidence of direct target engagement.

Across the selected target panel, AutoDock Vina scores showed a heterogeneous pattern (Figure 7D, Table 2); therefore, the scores were used to prioritize poses within this computational screen rather than to claim a uniform CAG advantage over AS-IV.CAG yielded scores of −10.41 kcal/mol in the PARP1 model and −9.48 kcal/mol in the AKT1 model. These values were used to select representative CAG–PARP1 and CAG–AKT1 poses for visualization and do not establish comparative binding affinity or pharmacological activity.

**Figure 8:**
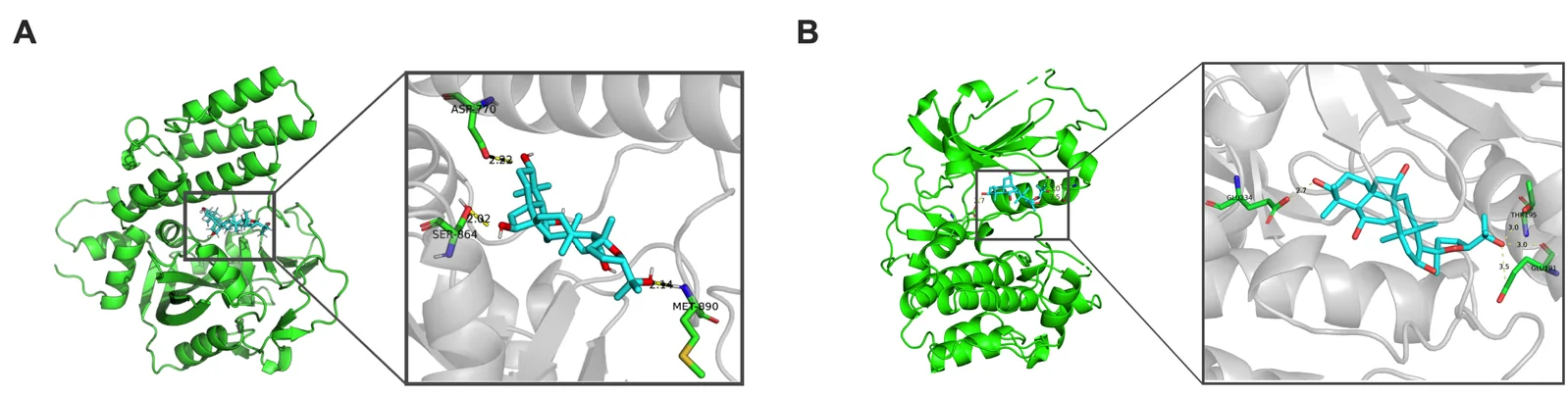
Representative CAG docking poses in the modeled PARP1 (**A**) and AKT1 (**B**) pockets, respectively. In the overall view, the protein is colored green and the ligand CAG in cyan. In the magnified insets, the pocket surface is shown in gray, interacting residues are highlighted in green and labeled in black. Predicted hydrogen-bond contacts are shown with dashed lines and distances in Å. These models are computational hypotheses and do not establish direct binding, target engagement, enzyme modulation, or causal contribution to the protective phenotype.

Inspection of the representative poses identified predicted hydrogen-bond contacts between CAG and Asp770 (2.22 Å), Ser864 (2.02 Å), and Met890 (2.14 Å) in the modeled PARP1 pocket (Figure 8A). In the modeled AKT1 pocket, CAG was predicted to contact Glu234 (2.7 Å), Thr195 (3.0 Å), and Glu191 (3.0 Å) (Figure 8B). These structural models offer testable interaction hypotheses; direct binding, target engagement, enzyme modulation, and causal dependence on PARP1 or AKT1 were not assessed in the present study.

## 4. Discussion

In this D-galactose-induced accelerated-aging model, oral CAG was associated with lower ABR thresholds, greater preservation of cochlear hair-cell architecture, and improvements in redox-, inflammatory-, and apoptosis-related indices. Comparative pharmacokinetic studies further showed greater systemic and perfused whole-cochlea exposure to CAG after direct CAG administration than after AS-IV administration. Together, these findings support continued preclinical evaluation of CAG while defining the model, tissue-distribution measurements, and computational analyses as important boundaries on mechanistic and translational interpretation.

The D-galactose model reproduced selected features relevant to auditory aging, including elevated ABR thresholds, hair-cell disorganization, oxidative stress, and inflammatory abnormalities. CAG treatment attenuated several of these outcomes, with the 20 mg/kg group generally showing larger changes than the 5 mg/kg group. The accompanying preservation of hair-cell architecture provides morphological support for the functional ABR findings. Nevertheless, this chemically induced accelerated-aging model should be regarded as an ARHL-like injury model rather than a complete surrogate for natural presbycusis [36,42,43]. These in vivo findings therefore establish preclinical activity under the present experimental conditions rather than definitive therapeutic efficacy in natural aging.

Achieving adequate inner-ear exposure remains an important challenge in neurotological drug development [6,7]. Under the tested dosing conditions, direct CAG administration produced greater plasma exposure and higher CAG concentrations in transcardially perfused whole-cochlea homogenates than AS-IV administration. This comparison supports a pharmacokinetic advantage of administering CAG directly, but it does not establish transport across the intact blood-labyrinth barrier or demonstrate exposure within a specific cochlear cell population [44,45].

While AS-IV has demonstrated bioactivity in various in vitro assays, our in vivo LC-MS/MS data showed lower intact AS-IV exposure in plasma and perfused whole-cochlea homogenates than the CAG exposure achieved by direct CAG dosing. Furthermore, our ex vivo mouse liver microsome assays demonstrated NADPH-dependent formation of CAG from AS-IV, supporting AS-IV as a metabolic precursor of CAG under these assay conditions. The same dataset also showed time-dependent loss of directly incubated CAG, indicating that CAG should not be described as microsomally stable in this system. This experiment does not establish the liver as the exclusive or quantitatively dominant conversion site, because gastrointestinal and other metabolic routes were not assessed [15,16,17,18]. Direct CAG dosing avoids the need for AS-IV-to-CAG conversion before CAG appears systemically and produced greater measured CAG exposure; however, the whole-cochlea data do not directly measure BLB permeability or establish pharmacologically active concentrations at a specific target site [6,44,45]. These exposure findings are consistent with, but cannot be causally linked to, the in vitro pharmacodynamic findings.

Oxidative stress and chronic low-grade inflammation are well-established contributors to auditory aging [1,2,3,4,5]. In the present model, CAG treatment was associated with lower serum concentrations of the pro-inflammatory cytokines TNF-α and IL-6 after D-galactose exposure.

In cochlear homogenates, CAG treatment was associated with higher SOD and GSH levels and lower ROS and MDA levels, while serum TNF-α and IL-6 concentrations were reduced. These concordant changes are consistent with attenuation of oxidative and inflammatory stress. However, the present measurements do not determine whether the systemic cytokine changes caused the cochlear effects, whether CAG acted directly within the cochlea, or which cochlear cell types contributed to the tissue-level signals.

Mitochondrial apoptosis is one pathway implicated in sensory-cell loss during auditory aging. Our in vitro and in vivo measurements showed CAG-associated changes in BAX, BCL2, and caspase-3 that were directionally consistent with reduced apoptosis. Together with the cellular ROS, mitochondrial-membrane-potential, and apoptosis assays, these results support an apoptosis-related interpretation. They do not, however, demonstrate that modulation of the BAX/BCL2/caspase-3 axis is necessary or sufficient for the observed auditory effects.

Systems pharmacology nominated SIRT1-related longevity signaling and apoptosis-associated proteins as candidate mechanistic links. This interpretation is biologically plausible because prior causal studies have connected cochlear SIRT1 signaling with oxidative stress, mitochondrial quality control, senescence, and hair-cell survival [24,25,26,27]. In the present study, however, SIRT1 expression or activity, direct target engagement, and dependence on SIRT1 were not measured. The updated docking panel showed heterogeneous scores across target models. CAG poses in PARP1 (−10.41 kcal/mol) and AKT1 (−9.48 kcal/mol) were selected for structural visualization. These model-dependent results prioritize PARP1 and AKT1 for direct biochemical and functional follow-up; they do not demonstrate binding affinity, PARP1 or AKT1 modulation, activation of SIRT1, or causal execution of the protective phenotype observed in vivo.

A growing number of plant-derived natural products and traditional Chinese medicines (TCMs) have recently been reported to prevent hearing dysfunction, and it is instructive to compare their efficacy with the present findings. The standardized Ginkgo biloba extract EGb761 attenuated aging-related caspase-mediated apoptosis in the rat cochlea, indicating that anti-apoptotic mechanisms contribute to the preservation of cochlear integrity during aging [46]. In an in vitro model of auditory senescence, cocoa flavonoids protected auditory senescent cells from oxidative-stress-induced apoptosis [47], in line with the ROS-scavenging and mitochondrial-protective effects of CAG observed here. The curcumin analog C1 delayed age-related hearing loss and sensory hair-cell injury in C57BL/6 mice by promoting TFEB nuclear localization and autophagy [48], whereas the coumarin derivative 5,7-dihydroxy-4-methylcoumarin attenuated cisplatin-induced ototoxicity by suppressing oxidative stress and apoptosis [49]. Notably, Erlong Zuoci decoction—the classical TCM formula used as the positive control in the present study—was previously shown to ameliorate age-related hearing loss in C57BL/6J mice [50], consistent with its protective effects in the D-galactose-induced model reported here. Taken together, these studies demonstrate that diverse plant-derived compounds and TCM formulas converge on shared protective pathways, including oxidative-stress suppression, mitochondrial stabilization, and apoptosis inhibition. Within this context, CAG offers a distinct pharmacokinetic advantage: as the aglycone of AS-IV, it achieves markedly higher plasma and cochlear exposure after oral administration, which may translate into more effective otoprotection at clinically relevant doses.

Several limitations define the scope of these conclusions. First, the D-galactose model captures selected oxidative and senescence-related features but does not reproduce the chronology or full pathology of natural auditory aging; confirmation in naturally aging models, such as aged C57BL/6J mice, is therefore needed. Second, separate one-way ANOVA at each frequency does not model the within-animal frequency structure and should be considered when interpreting frequency-dependent effects. Third, perfused whole-cochlea homogenates cannot resolve cochlear compartments, cell types, unbound concentrations, or BLB transport. Fourth, the efficacy and pharmacokinetic studies used different species, which limits direct exposure-response integration. Finally, the systems-pharmacology and docking analyses are hypothesis-generating; target-engagement, SIRT1-activity, inhibitor, knockdown, or rescue studies are required to test causality.

## 5. Conclusions

In summary, direct CAG administration increased systemic and perfused whole-cochlea CAG exposure relative to intact AS-IV after equivalent dosing—an advantage that persisted after molar-dose normalization—and attenuated auditory dysfunction, hair-cell injury, and redox-, inflammatory-, and apoptosis-related abnormalities in a D-galactose-induced accelerated-aging model. Because CAG and AS-IV showed comparable protective activity at equimolar concentrations in vitro, the principal advantage of CAG lies in its pharmacokinetic profile. These findings support further evaluation of CAG in natural-aging models. Systems pharmacology and molecular docking nominate DNA-damage-, survival-, and apoptosis-associated proteins as testable hypotheses; direct BLB transport, target engagement, enzyme modulation, and pathway causality remain to be established.

## Figures and Tables

**Table 1:** PK parameters of CAG and AS-IV in C57BL/6 mice.

Parameter	Unit	CAG Oral Group	AS-IV Oral Group	
		Analyte: CAG	Analyte: AS-IV	Analyte: CAG (Metabolite)
Plasma C_max_	ng/mL	1285.4 ± 162.5	155.2 ± 28.4	425.6 ± 55.8
Plasma T_max_	h	0.53	0.52	4.1
Plasma AUC_0–24h_	h·ng/mL	5642.8 ± 485.2	485.6 ± 75.2	3125.4 ± 350.6
Plasma t_1/2_	h	4.2 ± 0.8	1.8 ± 0.4	5.1 ± 0.9
Cochlea C_max_	ng/g	495.6 ± 72.1	26.8 ± 7.5	182.5 ± 31.2
Cochlea AUC_0–24h_	h·ng/g	1985.4 ± 215.6	45.2 ± 12.4	1050.8 ± 145.2
K_p_(Cochlea/Plasma)	Ratio	0.35 ± 0.04	0.09 ± 0.02	0.33 ± 0.05

**Table 2:** AutoDock Vina score of CAG and AS-IV with top 20 target protein.

No	Target Gene	Protein Name	PDB ID	AS-IV AutoDock Vina Score	CAG AutoDock Vina Score
				(kcal/mol)	(kcal/mol)
1	SIRT1	NAD-dependent protein deacetylase sirtuin-1	4I5I	−5.79	−6.86
2	BCL2	Apoptosis regulator Bcl-2	4LXD	−7.52	−8.56
3	CASP3	Caspase-3	1NME	−6.07	−6.33
4	BAX	Apoptosis regulator BAX	2LR1	21.26	−2.84
5	TP53	Cellular tumor antigen p53	1TUP	−4.26	−7.03
6	AKT1	RAC-alpha serine/threonine-protein kinase	3QKK	−9.70	−9.48
7	IL6	Interleukin-6	1ALU	−5.68	−7.52
8	TNF	Tumor necrosis factor	1TNF	−4.81	−6.52
9	RELA	Transcription factor p65 (NF-κB)	1NFI	−8.05	−7.75
10	PRKAA1	5′-AMP-activated protein kinase (AMPK)	5UFU	−7.61	−7.76
11	PIK3CA	Phosphatidylinositol 4,5-bisphosphate 3-kinase	4JPS	−6.55	−6.85
12	SOD1	Superoxide dismutase [Cu-Zn]	1HL5	−4.45	−6.48
13	NFE2L2	Nuclear factor erythroid 2-related factor 2 (Nrf2)	4IQK	−7.37	−7.99
14	VEGFA	Vascular endothelial growth factor A	1VPF	−6.83	−6.08
15	MAPK1	Mitogen-activated protein kinase 1 (ERK2)	4ZZN	−8.33	−7.85
16	STAT3	Signal transducer and activator of transcription 3	6NJS	−6.94	−6.95
17	PTGS2	Prostaglandin G/H synthase 2 (COX-2)	5F19	−7.66	−7.62
18	CASP9	Caspase-9	2AR9	−7.16	−7.57
19	PARP1	Poly [ADP-ribose] polymerase 1	4R6E	−9.70	−10.41
20	MTOR	Serine/threonine-protein kinase mTOR	4JT6	−9.10	−7.94

## Data Availability

Data will be made available upon reasonable request to the corresponding author.

## Abbreviations

The following abbreviations are used in this manuscript:

ABR	Auditory Brainstem Response
AgNO_3_	Silver nitrate
ANOVA	Analysis of variance
ARHL	Age-related hearing loss
AS-IV	Astragaloside IV
AUC	Area under the concentration–time curve
Bax	Bcl-2-associated X protein
BCA	Bicinchoninic acid assay
BCL2	B-cell lymphoma 2
BLB	Blood-labyrinth barrier
CAG	Cycloastragenol
CASP3	Caspase-3
CCK-8	Cell Counting Kit-8
CDFH-DA	2′,7′-Dichlorodihydrofluorescein diacetate
CLint	Intrinsic clearance
Cmax	Maximum concentration
CMC-Na	Sodium carboxymethyl cellulose
CO_2_	Carbon dioxide
D-gal	D-galactose
DAVID	Database for Annotation, Visualization and Integrated Discovery
DCFH-DA	2′,7′-Dichlorodihydrofluorescein diacetate
ECL	Enhanced chemiluminescence
ELISA	Enzyme-linked immunosorbent assay
GP	Erlong Zuoci Wan (positive control group)
GO	Gene Ontology
GSH	Glutathione
HEI-OC1	House Ear Institute-Organ of Corti 1
HSD	Honestly significant difference
IL-6	Interleukin-6
JC-1	5,5′,6,6′-Tetrachloro-1,1′,3,3′-tetraethylbenzimidazolylcarbocyanine iodide
KEGG	Kyoto Encyclopedia of Genes and Genomes
Kp	Cochlea-to-plasma partition coefficient
LC-MS/MS	Liquid chromatography–tandem mass spectrometry
MDA	Malondialdehyde
MFI	Mean fluorescence intensity
MMP	Mitochondrial membrane potential
MRM	Multiple reaction monitoring
NCA	Non-compartmental analysis
NADPH	Nicotinamide adenine dinucleotide phosphate
OMIM	Online Mendelian Inheritance in Man
PBS	Phosphate-buffered saline
PCR	Polymerase chain reaction
PDB	Protein Data Bank
PI	Propidium iodide
PK	Pharmacokinetic(s)
PPI	Protein–protein interaction
PVDF	Polyvinylidene fluoride
qPCR	Quantitative polymerase chain reaction
RCSB	Research Collaboratory for Structural Bioinformatics
ROS	Reactive oxygen species
RT-qPCR	Reverse transcription quantitative polymerase chain reaction
SD	Standard deviation
SDS-PAGE	Sodium dodecyl sulfate–polyacrylamide gel electrophoresis
SIRT1	Sirtuin 1
SOD	Superoxide dismutase
STRING	Search Tool for the Retrieval of Interacting Genes/Proteins
Tmax	Time to maximum concentration
TNF-α	Tumor necrosis factor-alpha
